# The Challenge Coping and Resilience of the Families of School-Aged Children with Autism Spectrum Disorder in China: A Qualitative Study

**DOI:** 10.3390/bs15040409

**Published:** 2025-03-23

**Authors:** Fengying Han, Xin Gao

**Affiliations:** 1 Department of Sociology and Social Work, School of Philosophy and Sociology, Northwest Normal University, Lanzhou 730070, China; 2 Social Work Department, School of Social Sciences, Universiti Sains Malaysia, Penang 11800, Malaysia

**Keywords:** challenges, coping processes, family resilience, school-aged children with autism spectrum disorder, China, qualitative study

## Abstract

This study aimed to explore the dynamic adjustment mechanisms of the families of school-aged children with autism spectrum disorder (ASD) in China in coping with challenges, focusing on the roles of belief systems, organizational processes, and communication strategies, as well as the influence of the China-specific cultural and policy contexts. Based on Walsh’s family resilience theory, a qualitative research methodology was used, with semi-structured interviews to collect experience data from these families, and thematic analysis was used to summarize the main challenges and coping processes. The study found that the families mainly faced the following challenges: difficulties in family care, parenting burnout, educational plights, and inadequate community support systems. Regarding belief systems, families enhanced their resilience through emotional acceptance and redefined expectations; regarding organizational processes, families optimized their internal operations through the flexible division of labor and decision-making patterns and actively mobilized external resources; and, regarding communication, reflection and sharing fostered emotional connection within the family, while compromise and patience enhanced the ability to integrate external resources. In addition, the traditional Chinese culture and inadequate policy support had a significant impact on the formation of family resilience. This study validates the cross-cultural applicability of family resilience theory and suggests enhancing family resilience through psychological support, policy optimization, and social advocacy.

## 1. Introduction

### 1.1. Autism Spectrum Disorder (ASD)

ASD is a neurodevelopmental disorder that primarily affects an individual’s social interactions, communication skills, and patterns of interest and behavior (American Psychiatric Association, 2013). The exact cause of ASD is not yet fully understood but is thought to be a combination of genetic and environmental factors (WHO, 2023). At the same time, a diagnosis of ASD is usually accompanied by a diagnosis of intellectual disability (American Psychiatric Association, 2013; Gao & Drani, 2024). Currently, there is no effective cure for ASD (WHO, 2023). The mainstream approach is long-term intervention training for individuals with ASD, and the intervention scenarios include families, schools (regular and special schools), intervention organizations, hospitals, etc. (Gao et al., 2023). The 2023 Census Report on Individuals with Disabilities in China, released by the China Disabled Persons’ Federation, reveals that more than 13 million individuals in the nation have been diagnosed with ASD. Within this group, roughly three million are children (0–18 years old) with ASD, and there is an annual addition of approximately 200,000 new cases (China Disabled Persons’ Federation, 2024). Currently, the prevalence of ASD is the highest among all mental disabilities in China (China Disabled Persons’ Federation, 2024). Furthermore, the escalating incidence rate has a ripple effect, manifesting in various challenges at the individual, familial, community, societal, healthcare, and social security levels.

### 1.2. Families of Children with ASD

Based on the specificity of the symptoms of ASD, the families of children with ASD need to provide lifelong care (Gao et al., 2023; Shorey et al., 2020). Several studies have demonstrated that the families of children with ASD experience stress from multiple sources, including psychological, physical, economic, familial relationships, social policies, and sociocultural factors, etc. (Bonis, 2016; Gülbetekin et al., 2024; Ha et al., 2014). Furthermore, the family caregivers of children with ASD typically experience higher levels of stress and lower levels of satisfaction in family life compared to the family caregivers of children with other developmental disabilities (e.g., Down syndrome, single intellectual disability, etc.) (Kuhlthau et al., 2014; Rivard et al., 2014). This is due to the complexity of the diagnostic process of ASD, as well as the complexity of the needs of children with ASD and the high variability in symptoms, as revealed during subsequent interventions and caregiving (Dawson et al., 2023; Turns et al., 2019). The impact of ASD on a family in China is characterized by significant social, cultural, and policy contexts (Feng et al., 2022; Gao et al., 2023; Tait et al., 2016). The concept of clans and “passing on the family line”, as well as the emphasis on the concept of collectivity and collective honor in Chinese culture, have led to the rejection of disability, which is seen as bringing shame to the whole family and even to the clan (Tait et al., 2016; Wang et al., 2011). Families may also experience a “loss of face” because of the uniqueness of ASD, and they may hide or ignore the needs of children with ASD (Ng & Ng, 2022). In addition, while the attention to ASD within the Chinese government as well as the healthcare sector has increased in recent years, the coverage of existing policies is still limited, particularly in the areas of support in school-age and adult individuals with ASD (Gao et al., 2023). Therefore, the family remains the main party responsible for the rehabilitation, education, and placement of children with ASD. They are generally under high financial pressure.

### 1.3. Resilience in Families of Individuals with ASD

From the perspective of ecosystem theory, the family is viewed as a dynamic, open system where individual members and subsystems are interconnected and work together (Maltby et al., 2019). Family resilience, within the framework of ecosystem theory, can be understood as families’ ability to cope with crises or challenges through adaptation, integration, and interaction with the external environment (De Haan et al., 2002; Henry et al., 2015; Walsh, 2015, 2021; West et al., 2011). This theory refutes previous research on families based on the “deficit perspective” (Walsh, 2021). Research has shown that the families of children with ASD typically have lower levels of resilience compared to the families of normally developing children (Bayat & Schuntermann, 2013; Hashimoto, 2020; Hassanein et al., 2021; Sardohan Yildirim et al., 2025). It has also been shown that adjustments in the three dimensions of family beliefs, organizational processes, and communication strategies significantly affect the ability of the families of children with ASD to cope with challenges and can enhance their ability to actively seek social support (Gao et al., 2023). Having good resilience helps the families of children with ASD to accept their child’s situation relatively smoothly and actively learn the skills needed to cope with challenges (Hassanein et al., 2021). Having good resilience not only helps these families to establish positive connections with the outside world but also plays a key role in reducing their stress (Bayat & Schuntermann, 2013).

### 1.4. Current Research

There is now growing academic interest in the families of children with ASD. Areas of concern are related to family intervention, social support, stigma, and family quality of life, etc. (Gao et al., 2023; Gao & Drani, 2024; Hashimoto, 2020; Hassanein et al., 2021; Sardohan Yildirim et al., 2025). However, there is still room for further exploration. First, most of these studies have focused on the families of children with ASD aged 0–7 years, i.e., preschool children, as well as focusing on the various challenges of these families during their child’s diagnosis (Gao et al., 2023; Shattnawi et al., 2021). Alternatively, some studies lack clarity regarding the ages of individuals with ASD. It is worth noting that the challenges faced by their families change with different developmental stages (Ilias et al., 2019). It has been proposed that, for children with ASD, the stage of school age is a peak period of stress for their families, especially in terms of educational choices and social adjustment (Cole et al., 2017; Manning et al., 2011). Therefore, there is a need for an in-depth discussion of the challenge coping process in the families of school-aged children with ASD. Second, current research on resilience in the families of individuals with ASD tends to view resilience as a mediating or protective factor, and the research methods tend to be quantitative; thus, these studies mostly focus on the validation of specific relationships (Gao & Drani, 2024; Sardohan Yildirim et al., 2025; Yang et al., 2018). There is a lack of research that integrally analyzes the life experiences of the families of individuals with ASD through the lens of family resilience. Third, the formulation and evolution of family resilience theory are deeply rooted in the social and cultural contexts of Western countries. This contextual foundation is a critical factor that must be considered when examining family resilience. Variations in sociocultural settings significantly influence how families respond to challenges and manifest resilience (Walsh, 2015, 2021). However, the adaptation of family resilience theory within Chinese academia remains underdeveloped, leaving considerable scope for further exploration in this area. Conducting in-depth research on the impact of China’s unique social and cultural contexts on family resilience will not only enhance the theoretical framework but also provide more precise guidance for practical applications.

### 1.5. Theoretical Framework and Research Questions

**Figure 1:**
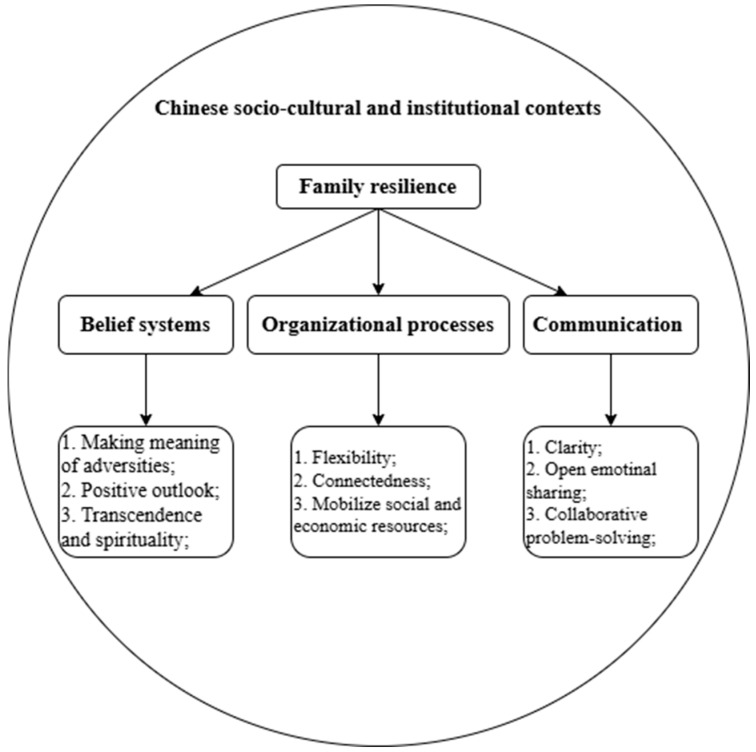
Theoretical analysis framework.

This study takes the families of school-aged children with ASD in China as the research population and analyzes the challenge coping processes of these families through the analytical framework of family resilience theory. A qualitative research method is used in this study. This study refers to the theoretical analysis framework of family resilience proposed by Walsh (2015, 2021) and analyzes the challenge coping processes of these families in terms of three dimensions: belief systems, organizational processes, and communication (refer to Figure 1). At the same time, this study notes the impact of China’s particular sociocultural and institutional contexts on the generation of resilience in these families. Therefore, the objective of this study is to explore the challenge coping experiences of the families of school-aged children with ASD in China with an integrated, strength-based perspective. In summary, the research questions are as follows: (1) What are the challenges faced by the families of school-aged children with ASD in China? (2) How do these families demonstrate resilience in coping with their challenges?

## 2. Method

### 2.1. Research Design

The Consolidated Criteria for Reporting Qualitative Research (COREQ) were used to guide this study (Tong et al., 2007). This study used the interpretive phenomenological analysis approach (Peat et al., 2019; Smith & Osborn, 2015) to explore the main challenges faced by the families of school-aged children with ASD and their challenge coping processes in China. This approach is appropriate because it emphasizes the lifeworld of the research participants, focusing on how they perceive and interpret the phenomena that they experience, with the goal of revealing the core essence of the phenomena, rather than merely describing their surface features (Peat et al., 2019). Therefore, the researcher is able to deeply analyze the real-life experiences and emotions of the research participants.

### 2.2. Research Participants

The inclusion criteria for participants in this study were as follows: (1) individuals with ASD who were of school age (7–18 years old) (note: school age was defined with reference to the Law of the People’s Republic of China on Compulsory Education and the Law of the People’s Republic of China on the Protection of Minors) (Central People’s Government of the People’s Republic of China, 2020, 2021); (2) individuals with a diagnosis certificate from an organization with statutory qualifications for the diagnosis of ASD; (3) the immediate family members of individuals with ASD, i.e., parents and grandparents; (4) complete caregiving experience (from the time of diagnosis to the present); and (5) a relatively high level of family resilience. The exclusion criteria were as follows: (1) those with significant physical or psychological illnesses; (2) those who expressed a negative attitude towards participating in the study; and (3) those who were unable to ensure their full participation.

This study used purposive sampling. This study was supported by the Fifth Hospital of Yulin City, Shaanxi Province, China. This hospital is a public hospital specializing in the diagnosis and treatment of various neurodevelopmental disorders. Several ASD rehabilitation trainers from the hospital recommended families to the researchers who met the inclusion criteria. The researchers then sent invitations to these families to participate in this study. Recruitment was conducted by the first author (female; PhD). The first author’s area of specialization was social psychology, with a long-term focus on the families of individuals with ASD, and they had independently completed a number of qualitative studies and had relevant professional competencies.

Regarding the aspect of confirming whether the potential participants had a relatively high level of family resilience, while referencing the recommendation information from the ASD rehabilitation trainers, the researchers also used the Family Resilience Scale Short Form (Chinese version) (FRS16-C) for reconfirmation (Chow et al., 2022). The FRS16-C contains 16 items that can be divided into three dimensions: communication and connectedness, positive framing, and external resources (Chow et al., 2022). The scale is a 4-point scale (1 = strongly disagree to 4 = strongly agree), and the FRS16-C has been validated to have satisfactory reliability and validity (Chow et al., 2022). Moreover, the design of the scale was based on Walsh’s family resilience theory, which aligned well with this research (Chow et al., 2022). The researchers compared each potential participant’s scores to normative data through a one-sample *t*-test. Potential participants whose scores were not significantly different from or significantly higher than the normative data were considered eligible. The normative data were derived from a survey of 1135 general Chinese people by Chow et al. (2022) (using the same scale). Ultimately, 16 family caregivers of school-aged children with ASD from 8 families participated in this study. Of these, 9 were female and 7 were male; 6 were mothers, 6 were fathers, 3 were grandmothers, and 1 was a grandfather; the age distribution was 32–59 years old. Detailed information is provided in Table 1. In addition, the participants’ scale scores were not significantly different from the normative data according to the one-sample *t*-test (*p* > 0.05).

### 2.3. Data Collection

The data collection method for this study was a semi-structured interview. Semi-structured interviews combine a framework and flexibility to ensure core topic coverage while offering insights into individual experiences to obtain rich qualitative data (Dearnley, 2005; Kallio et al., 2016). They are suitable for exploring complex or sensitive topics, such as the resilience of the families of individuals with ASD, providing authentic and diverse research perspectives (Hosseinpour et al., 2022; Ng & Ng, 2022). Prior to data collection, two researchers designed an interview outline based on the research questions, a literature review, and the theoretical analysis framework, and a pilot study was implemented to identify any vague or potentially misleading questions (In, 2017; Kunselman, 2024; Thabane et al., 2010). The participants in the pilot study were not involved in the formal study, and the data collected in the pilot study were encrypted in line with the participants’ consent. A detailed interview outline can be found in the Supplementary Materials.

After the participants were fully informed about this study (information about the researchers, research objectives, research content, research period, etc.) and signed the informed consent form, the second author started the data collection work. The second author (male; student enrolled in a doctoral program) had obtained Bachelor’s and Master’s degrees in social work, had a longstanding interest in the families of individuals with ASD, and had independently completed a number of qualitative studies and had relevant professional competencies. All interviews were one-to-one and conducted offline. All interviews were conducted in Chinese. The study site was the participant’s home or a cafe with a relatively high level of privacy. Each interview lasted 60–80 min. All interviews were audio-recorded with informed consent. In addition, all process notes (schedules, researchers’ reflections) for this study were in the form of electronic text and were stored in an encrypted storage device together with the audio files to ensure data security.

### 2.4. Data Analysis

After a total of 35 interviews, the researchers determined that data saturation had been achieved (Faulkner & Trotter, 2017), and they ended the data collection stage and began data analysis. This study used thematic analysis for the data analysis work (Clarke & Braun, 2018; Mihas, 2023). The NVivo qualitative data analysis software was used for data management.

First, the two authors transcribed the audio recordings into electronic text through the intelligent transcription function of the audio recording device. The two authors checked the consistency of the textual content and the content of the recordings and then translated the text from Chinese to English. Codes were assigned to all participants for de-identification. The transcription and translation processes were overseen by all participants. Each participant was granted access to review only the recordings (including transcripts and translations) that they themselves had provided at any time. Secondly, the two authors imported the transcripts into the NVivo 12 software and conducted preliminary data sorting through three methods: word frequency, synonyms, and automatic node generation. Third, the two authors independently coded the data line by line based on the three dimensions of beliefs, organization, and communication. Then, the two authors compared the coding results, discussed consistencies and discrepancies, and adjusted or combined the codes if necessary. Third, the second author generated initial themes based on the coding and matched them to the established framework. The first author then reviewed whether the themes adequately covered the data content and proposed additions or adjustments to unclear themes. Finally, the two authors reviewed the themes together to ensure that each theme matched the data and research questions; they then checked to ensure that there were no overlapping or redundant themes and merged or redefined the themes if necessary; and they identified the names and definitions of each theme and selected key data segments to support them as examples.

## 3. Results

**Figure 2:**
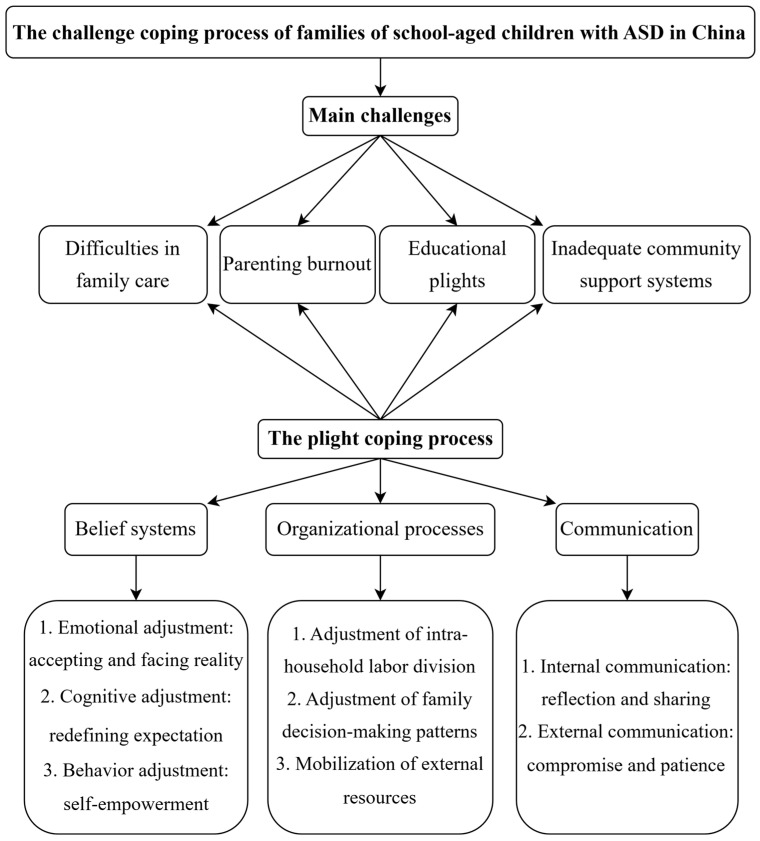
Main results.

The results of this study are divided into two parts: (1) the main challenges of the families of school-aged children with ASD in China and (2) the challenge coping process based on the family resilience perspective. The detailed thematic structure is shown in Figure 2.

### 3.1. Main Challenges

#### 3.1.1. Difficulties in Family Care

First, all participants reported that ASD did not significantly affect their child’s physical development. Additionally, they reported that, during the school-age period, their children’s height, weight, and strength increased significantly; therefore, their children’s emotional and behavioral problems became more difficult to control effectively, making family care more difficult. Second, all participants reported difficulties in arranging diets for their children with ASD during the school period. Because they were unable to accurately express their needs, some of their diet-related problems, such as picky eating, anorexia, and food intake, could not be accurately assessed by their families, which could lead to insufficient or unbalanced nutrient intake, thus affecting their growth and development. There were also eight participants (1-1, 2-2, 3-1, 3-2, 4-1, 6-2, 7-1, and 8-2; one father, four mothers, and three grandmothers) who mentioned their children’s toileting, sexual development, and sleep disorders, which also become more difficult to deal with as their bodies developed rapidly.

*“I don’t know how much of each meal will fill him up, and several times he’s eaten until he vomits. Also, he’s growing taller and taller, and there’s no way I can control him effectively by myself anymore when he’s angry”.*

(1-1)

#### 3.1.2. Parenting Burnout

All participants reported that they experienced parenting burnout from time to time. First, they felt physically and mentally exhausted by the prolonged and intense caregiving tasks and lacked the energy to cope with daily life. Second, they became skeptical of their own parenting abilities, believing that they were unable to effectively meet their children’s needs. Third, due to continuous stress and fatigue, they could develop negative attitudes towards child care and even emotional detachment. In addition, the traditional Chinese fertility culture impacted them. This was particularly evident in the grandparent generation. Four participants (3-2, 4-2, 6-2, and 8-2; three grandmothers and one grandfather) viewed child-rearing as an important responsibility for family heritage and cultural continuity, and they were particularly sensitive to the burnout and stress that occurred in the child-rearing process. Parenting burnout, in turn, can lead to family conflict and even negatively impact children’s emotional and behavioral management.

*“I sometimes just have a deep sense of powerlessness, feeling abandoned and isolated. The whole person is drained. When I come home from work, I don’t want to take care of the child or talk to anyone else in the family”.*

(3-1)

*“Passing on the family line is of course important! I’ve told them many times to have another child while I’m still healthy and I’ll take care of him or her”.*

(8-2)

#### 3.1.3. Educational Plights

China is currently implementing an integrated education policy for school-aged children with developmental disabilities. However, according to the feedback from all participants, there are fewer resources (equipment, teachers) for special education in ordinary schools, and six participants (2-1, 3-1, 4-2, 5-1, 7-2, and 8-1; five fathers and one grandfather) indicated that their children had been refused enrollment in some ordinary schools. This makes it difficult to meet the individualized needs of children with ASD. At the same time, children with ASD may face ostracism or discrimination by their peers in ordinary schools, affecting their social adjustment and mental health. In addition, the number of special education schools is far from meeting the real needs. Nine participants (1-1, 1-2, 3-1, 3-2, 4-1, 4-2, 5-1, 7-1, and 7-2; two mothers, five fathers, one grandmother, and one grandfather) stated that they had undergone the process of renting rooms, relocating, and operating interpersonal relationships across counties for the sake of their children’s rehabilitation education. This led to varying degrees of economic stress for their families.

*“There is no special education school in my county, and I have contacted several ordinary schools and they all refused to enroll my child. So, I had to ask relatives and friends about schools in other places, and it took me months to settle on coming to the current school. In response, my wife and I have moved and looked for new jobs”.*

(7-2)

#### 3.1.4. Inadequate Community Support Systems

In China, a community is defined as a self-governing organization of grassroots residents with the functions of management, service, social security, education, and safety and stability (Xiong & Li, 2024). However, all participants indicated that their own communities lacked support services for the population with developmental disabilities and their families, such as respite services, counseling, and family intervention training sessions, etc. At the same time, community health centers lack specialized equipment and medical staff to provide interventions for school-aged children with ASD. In addition, it is difficult for children with ASD to establish normal peer relationships with other children in the community, and they may experience rejection or neglect. Community activities are also often not designed with consideration of the unique needs of children with ASD, making it difficult for them to participate.

*“The community service center provides my child with an annual allowance of 500 RMB, and other than that, there is no other coverage. That’s not even enough money to cover half a month’s expenses for my child. My child sometimes has diarrhea and fever, and the community health center doesn’t dare to diagnose my child and tells us to go to a big hospital. The help they give us is very limited and not enough to relieve the pressure of us parents”.*

(8-1)

### 3.2. The Challenge Coping Process

#### 3.2.1. Belief Systems

##### Emotional Adjustment: Accepting and Facing Reality

All participants reported that, at school age, they had become more clearly aware of their child’s gaps in learning, socialization, and daily life, which could trigger feelings of anxiety, disappointment, and even self-blame. However, with a deeper understanding of their child’s condition and through interactions with other families of individuals with ASD, they came to recognize their child’s specific needs and began to accept their child’s uniqueness. This is the first step for these families to cope with their challenges and is an important starting point in replanning family life.

*“The older the child gets, the more obvious some of the behavioral problems become. It doesn’t do us any good to keep fluke mind, it only deepens the pain. Instead, I have a sense of relief after truly recognizing and accepting that my child is different”.*

(6-1)

##### Cognitive Adjustment: Redefining Expectation

Ten participants (1-1, 1-2, 2-1, 2-2, 3-1, 4-1, 5-2, 6-1, 7-2, and 8-1; five mothers and five fathers) reported that they came to realize that the traditional pathways to success (e.g., excelling academically and attending prestigious schools) may not apply to their children. They revised their expectations for their children, redirecting their focus towards enhancing their children’s well-being and developing practical life skills. This process is often accompanied by a deeper understanding of the boundaries of such children’s abilities, and family caregivers learn to appreciate their children’s uniqueness and realize their potential in specific areas, such as demonstrating a high level of concentration in carrying out certain tasks. Through this cognitive adjustment, families are able to set realistic goals to support their children’s long-term development and the balance of family life.

*“It is not fair to the child or to us to continue to have great ambitions for our child. Holding on to such ideas will only lead to tragedy. We are content as long as our child is healthy and suffers no other pain. We’ll be there for him”.*

(4-1)

##### Behavior Adjustment: Self-Empowerment

Twelve participants (1-1, 1-2, 2-1, 2-2, 3-1, 4-2, 5-1, 5-2, 6-1, 6-2, 7-1, and 8-2; five mothers, four fathers, two grandmothers, and one grandfather) indicated that they had started to take the initiative to access knowledge and skills, such as learning about family intervention methods, behavior management strategies, and educational concepts for ASD. Such learning enabled them to understand their children’s behavioral patterns and developmental potential, thus breaking down the negative perceptions formed by helplessness and anxiety. At the same time, through exchanging experiences with other family caregivers of individuals with ASD through the internet or offline, as well as contact with positive cases, all participants reported that they gradually developed the positive belief that “children can grow through effort and support”. This positive belief not only helped them to accept reality and redefine the standard of success but also strengthened their resilience in the face of social prejudice and educational plights, so that they gradually learned to care for themselves, reduce their stress, and shape a positive family atmosphere, thus transferring this positive belief to family members. Ultimately, this self-empowerment brings about a shift in beliefs that enables such individuals to cope with challenges and lays a solid foundation for the long-term adaptation of the family.

*“My child has ASD and there is no way for him to go through life like a normal child. But I won’t give up, I will do my best to support his rehabilitation, even if the progress is not obvious, I accept it. I learned some family intervention methods online and, by now, have been sticking to family intervention for 5 years. As a parent of a child with ASD, I think it’s the right thing to do. As long as he can stay healthy, I will be satisfied”.*

(2-1)

#### 3.2.2. Organizational Processes

##### Adjustment of Intra-Household Labor Division

All participants reported that their intra-household labor division was adjusted so as to more effectively share caregiving responsibilities and cope with complex needs. Mostly, one parent focused on the child’s rehabilitation and daily management, while the other parent was responsible for maintaining the family’s financial income. At the same time, other family members (e.g., grandparents) also joined the support system to take up some of the caregiving tasks. Through the clear division of responsibilities, these families can not only relieve the parenting pressure of a single member but also improve the overall operational efficiency of the family. In addition, during the adjustment process, six participants (2-1, 3-2, 4-1, 6-1, 7-2, and 8-1; two mothers, three fathers, and one grandmother) indicated that they were ensuring the fairness and sustainability of the intra-household labor division through regular communication and consultation. This dynamic adjustment of the intra-household labor division enables these families to develop a more stable and flexible organizational structure in the face of challenges, thus better supporting their children’s growth and coping with external challenges.

*“It used to be that both kids were working and I was taking care of my grandson all by myself. Now, my grandson has grown up and I was unable to take care of him on my own. So, my daughter-in-law has taken the initiative to adjust her job, earning less each month but spending more time at home. She has given a lot to this family”.*

(3-2)

##### Adjustment of Family Decision-Making Patterns

All participants mentioned that there had been an adjustment in the decision-making patterns within their families in order to cope with the complex educational and developmental needs of their children. This was often characterized by a shift from single or authoritative decision-making among family members to a more democratic and collaborative pattern. For example (from participant 5-2; one mother), couples may jointly decide on their child’s rehabilitation program, school choice, or intervention strategies through regular discussions that incorporate their own experiences and insights. Additionally, families may invite grandparents or professionals to participate in major decisions to bring in additional perspectives and support (from participant 8-2; one grandmother). Such an adjustment not only improves the scientific and rational nature of decision-making but also enhances the cohesion and adaptability within the family, so that it can respond more effectively to external challenges and the dynamic needs of the growing child.

*“In the past, the children’s father and grandfather used to make all the decisions in the family. Now, when there is something important in the family, we discuss it together and then make decisions”.*

(5-2)

##### Mobilization of External Resources

All participants reported that they sought external support through a variety of means. Eight participants (1-1, 1-2, 2-1, 3-1, 3-2, 4-2, 5-1, and 8-1; one mother, five fathers, one grandmother, and one grandfather) actively contacted professional organizations for rehabilitation training and special education services or worked with doctors and ASD rehabilitation trainers to develop individualized family intervention plans. Ten participants (1-2, 2-1, 2-2, 3-1, 4-1, 5-1, 5-2, 7-1, 7-2, and 8-1; four mothers and six fathers) joined parent support organizations or public welfare agencies to share their experiences and build emotional support networks, from which they could obtain practical advice and psychological strength; in addition, through the platforms of such organizations or agencies, they actively communicated with schools and teachers in order to participate in the allocation of educational resources and classroom adaptation. Regarding policy resources, all participants were able to engage by proactively understanding the relevant welfare policies and taking the initiative to apply for subsidies and insurance provided by the government in order to alleviate their financial pressure. By integrating these external resources, these families were able to obtain support at various levels, including emotional, financial, and educational, so as to better meet their children’s developmental needs and, at the same time, enhance the resilience of the family.

*“I’m too weak on my own. I joined an organization full of parents or grandparents etc. of children with ASD. We joined together to give advice to the Education Bureau, and they accepted it very quickly. Moreover, there are some government subsidies that many families wouldn’t know about if it wasn’t for the sharing of organization members”.*

(7-1)

#### 3.2.3. Communication

##### Internal Communication: Reflection and Sharing

Nine participants (1-1, 3-1, 3-2, 4-2, 5-2, 6-2, 7-1, 7-2, and 8-2; three mothers, two fathers, three grandmothers, and one grandfather) mentioned that they reflected and shared in the communication process with other family members. Through reflection, they and other family members were able to summarize their experiences and shortcomings in the parenting process, such as exploring the effectiveness of certain intervention strategies or identifying misunderstandings in daily communication. Sharing provides a channel for family members to communicate emotionally, enabling them to express their stress, confusion, and expectations openly to each other. Such interactions help families to form a stronger emotional bond and avoid the disconnection caused by the stress of long-term caregiving. At the same time, by sharing their successes and positive emotions, family members can work together to increase their confidence in the future and inspire teamwork. Reflection and sharing are not only means of adjusting internal relationships but also an important process of seeking solutions to challenges, providing a sustained impetus for the enhancement of family resilience.

*“I do self-reflection if I do something wrong. We have a hard enough time as a family. I communicate with my family if I am unhappy or happy about something, all to protect our family”.*

(7-2)

##### External Communication: Compromise and Patience

In the face of external challenges such as insufficient social support and limited educational resources, all participants indicated that they often used the communication strategy of compromise and patience to gradually build up cooperative relationships. When interacting with schools, healthcare organizations, or relevant government departments, they usually found mutually acceptable solutions through compromise. For example (from participant 2-1; one father), regarding the issue of school adaptation, one participant indicated that they might lower their expectations regarding the curriculum or teacher qualifications, while patiently communicating with the school about their child’s specific needs and striving for more practical adjustments. In terms of medical and policy support, all participants reported that they were often flexible in adjusting their expectations, seeking services that were appropriate for their children through continuous trial and feedback. This form of patience and compromise not only helps to reduce external conflicts but also earns more trust and support for the family and even the whole group, thus gradually improving the external environment and creating more favorable conditions for their children’s growth.

*“Staying tough is not an option. We must remain patient enough and make appropriate compromises. We cannot expect all these security and welfare benefits to be provided in one step, and we have to take our time”.*

(2-1)

## 4. Discussion

Based on family resilience theory, this study delves into the coping processes of the families of school-aged children with ASD in China in the face of multiple challenges. The study reveals how families achieve challenge alleviation and resilience building through dynamic adjustments in three dimensions: belief systems, organizational processes, and communication strategies. In this section, the core findings are further analyzed in the context of the research findings and discussed from the perspectives of sociocultural, policy support, and theoretical implications.

This study found that families underwent a dynamic process from emotional acceptance to redefining their expectations in terms of their belief systems. Many families showed emotional helplessness and anxiety when initially confronted with their children’s academic and social challenges, but, through learning about ASD and interacting with other families, they gradually recognized their children’s uniqueness and adjusted their expectations for the future. This shift in families, from emphasizing traditional pathways to success (e.g., academic excellence, ability to live independently) to focusing on their children’s daily living skills and well-being, is critical to the stability of the family as a whole (Sardohan Yildirim et al., 2025). This belief adjustment is not only a spontaneous process within the family but also relies on external support such as counseling, community education, and the dissemination of positive cases. In Chinese culture, the notion of “family honor” places additional pressure on families, and some families may find it more difficult to accept their children’s unique characteristics due to social prejudice (Gao et al., 2023; Tait et al., 2016). Therefore, society should promote positive changes in the belief system from the outside by increasing the dissemination of knowledge about ASD and eliminating social stigmatization.

Families enhance their ability to cope with challenges at the organizational level by adjusting the internal division of roles, optimizing decision-making patterns, and mobilizing external resources. This study found that most families adopted the more flexible division of labor—for example, one parent focused on caregiving and rehabilitation tasks, while the other parent was responsible for financial support. In addition, the families gradually developed a more democratic decision-making pattern, taking more advice from grandparents or professionals when addressing their children’s educational choices, rehabilitation programs, etc. This study highlights the importance of external resources for family organizational adaptation. However, inequality in resource access cannot be ignored—for example, urban families usually have easier access to high-quality education and rehabilitation resources, while rural families are constrained by a lack of resources and face greater pressure. At the policy level, the government should promote the balanced distribution of resources—for example, by increasing the number of special education schools and rehabilitation centers in rural areas and by simplifying the application process for related subsidies and services to make it less difficult for families to access resources.

Communication is an important tool for families to cope with challenges (Walsh, 2015, 2021), covering both reflection and sharing within the family and compromise and patience with the external environment. This study found that, through reflection, family members were better able to summarize their experiences, improve their strategies, and increase the trust and support in their emotional communication. At the same time, the families showed compromise and patience in their communication with schools, healthcare organizations, and policy departments, and, through active negotiation, they gradually strived for resources and services suitable for their children. This study suggests that communication is the first step in improving the external supportive environment of the family, but the long-term effects depend on the establishment of systematic cooperation. For example, schools should provide regular home-school meetings, and healthcare organizations should set up parental feedback mechanisms to support family participation and collaboration at the institutional level. In addition, the optimization of communication strategies within families requires more professional guidance, such as the provision of parent training courses to help families to establish efficient communication patterns (Altiere & von Kluge, 2009; Summers et al., 2007).

Additionally, although the analysis predominantly emphasized the integrated family unit, subtle variations in coping attitudes and strategies emerged across different family roles. For instance, parents generally exhibit a pragmatic orientation by actively engaging in problem-solving, mobilizing external resources, and adjusting intra-household roles to manage daily challenges. In contrast, grandparents often rely on traditional cultural values and provide emotional anchorage, reflecting a more passive yet supportive approach to coping. At the same time, nuanced gender differences are evident, with mothers frequently prioritizing emotional communication and relational maintenance, whereas fathers tend to focus on strategic decision-making and resource management. These role-specific insights contribute to a deeper understanding of family resilience and underscore the need for further research to disentangle these differences for the development of targeted interventions for the families of children with ASD.

The results of this study highlight the profound impact of China’s unique sociocultural and institutional context on the challenge coping processes of families. On the one hand, the traditional cultural concept of emphasizing family responsibilities (Cheng et al., 2021) provides intrinsic motivation for families, but it also increases the psychological burden on families. On the other hand, the lack of policy support and social services places families at a disadvantage when faced with high rehabilitation costs and the unequal distribution of resources. The government needs to strengthen its support for integrated education, community-based rehabilitation, and financial subsidies for families and to ensure that its policies can truly benefit families in need by improving the efficiency and transparency of their implementation. At the cultural level, the public understanding and acceptance of ASD should be enhanced through social publicity and education, so as to create a more friendly social environment for families.

## 5. Conclusions

This study shows that the families of school-aged children with ASD in China gradually achieve coping with challenges and the construction of family resilience through the adjustment of their belief systems, the optimization of organizational processes, and the multi-level application of communication strategies. These adjustment processes not only reflect the adaptive capacity of families but are also deeply influenced by the sociocultural and institutional contexts. Enhancing family resilience requires a multi-level approach, including the provision of psychological support, strengthening the supply of resources, optimizing the implementation of policies, and eliminating social prejudices. By integrating multiple efforts from the family, society, and policy, the development and integration of school-aged children with ASD and their families can be better supported, thus laying the foundation for the overall progress of society towards a more inclusive one. Future research needs to further explore the diverse coping strategies of families with different backgrounds and provide more targeted policy recommendations to promote the joint development of theory and practice.

## 6. Theoretical Contributions

This study extends the cultural applicability of Walsh’s family resilience theory by applying it to the challenge coping processes of the Chinese families of school-aged children with ASD. The study reveals the central role of belief systems in family adaptation, especially the fact that families promote the optimization of internal and external coping strategies by redefining their expectations. At the same time, the study highlights the dynamic nature of organizational processes and the multi-level role of communication strategies in internal emotional bonding and external resource integration. These findings enrich family resilience theory, provide new perspectives for cross-cultural research, and provide a theoretical basis for the design of related support services.

## 7. Research Limitations

The sample of this study focused mainly on families with higher resilience, which may not fully reflect the challenges of resource-poor families; the study design was cross-sectional, which failed to reveal the developmental dynamics of family resilience; and the analysis of the specific roles of traditional Chinese culture and policy support in family resilience is still insufficient. Future research could be further improved through a longitudinal design and broader sample coverage. In addition, comparative analyses of different family members are an important and valuable line of research. However, this study focused on the family as a whole, rather than individual differences in coping strategies. Future research could further explore the different experiences and perspectives of different family members to gain a more nuanced understanding of family resilience.

## Figures and Tables

**Table 1:** Demographic information of participants.

Family No.	Family Structure	Age of Individual with ASD	Participant No.	Age	Gender	Kinship	Educational Level	Scale Score	M (SD)	*t*-Value
1	Core family	10	1-1	35	Female	Mother	Secondary school	46	49.25 (2.86)	−0.223
			1-2	37	Male	Father	Secondary school	49		
2	Extended family	16	2-1	42	Male	Father	Bachelor’s degree	48		
			2-2	46	Female	Mother	Secondary school	50		
3	Extended family	14	3-1	39	Male	Father	Bachelor’s degree	45		
			3-2	59	Female	Grandma	Primary school	49		
4	Extended family	12	4-1	35	Female	Mother	Secondary school	51		
			4-2	57	Male	Grandpa	Primary school	49		
5	Core family	9	5-1	34	Male	Father	Primary school	53		
			5-2	38	Female	Mother	Secondary school	51		
6	Extended family	9	6-1	32	Female	Mother	Bachelor’s degree	45		
			6-2	51	Female	Grandma	Primary school	47		
7	Extended family	13	7-1	39	Female	Mother	Secondary school	55		
			7-2	39	Male	Father	Bachelor’s degree	51		
8	Extended family	11	8-1	34	Male	Father	Bachelor’s degree	52		
			8-2	55	Female	Grandma	Secondary school	47		

## Data Availability

The data presented in this study are available on reasonable request from the corresponding author, with the consent of all participants in this study.

## Supplementary Materials

The following supporting information can be downloaded at: https://www.mdpi.com/article/10.3390/bs15040409/s1, File S1. Interview Outline; File S2. Consolidated Criteria for Reporting Qualitative Studies (COREQ): 32-Item Checklist.
