# Supplementary material for: The Challenge Coping and Resilience of the Families of School-Aged Children with Autism Spectrum Disorder in China: A Qualitative Study
Source: Behav Sci (Basel). 2025 Mar 23;15(4):409. doi: 10.3390/bs15040409 (PMC12024200; doi:10.3390/bs15040409)
Supplement: Supplementary file 1 [file behavsci-15-00409-s001.zip › behavsci-3450282-supplementary.pdf]

File S1.

Interview Outline (English Version)

Introduction:

Thank you for participating in this interview. The purpose of this conversation is to explore the challenges that your family has faced and the ways that you have coped with them from the perspective of family resilience. Please feel free to share your thoughts and experiences openly. All information will be kept strictly confidential and used solely for academic research.

I. Family Belief Systems

1. **Making Meaning of Adversity**

How do you perceive your child's autism diagnosis? What does it mean to you and your family?

How do you and your family make sense of the challenges you face?

2. **Positive Outlook**

When encountering difficulties in raising your child, what keeps you and your family hopeful?

Do you or your family have any motivating beliefs or stories that inspire you to overcome challenges?

3. **Transcendence and Spirituality**

Do you or your family rely on certain beliefs, cultural values, or traditions to cope with adversity?

Have traditional Chinese cultural values (e.g., filial piety, family unity) played a role in your coping process?

II. Organizational Processes

1. **Flexibility and Adaptability**

How does your family adjust and adapt when facing significant changes in your child's education or daily life?

How do you assign family roles (e.g., caregiving, financial support) to handle these challenges?

2. **Connectedness**

How has the relationship between family members changed as you cope with difficulties?

How do you and your family support one another? Do you rely on extended family, friends, or community networks?

3. **Resource Mobilization**

Have you sought support from external resources (e.g., government assistance, school support, psychological services)? How have they helped?

Have you experienced difficulties accessing these resources? How did you cope with such challenges?

III. Communication / Problem-Solving Processes

1. **Communication Patterns**

How does your family typically discuss matters related to your child? Do these discussions help you manage problems?

Have communication issues ever worsened challenges? How did you resolve them?

2. **Emotional Sharing and Acceptance**

How do you and your family handle negative emotions (e.g., frustration, anger, anxiety)? Are these emotions openly expressed?

Do family members encourage one another to share their feelings?

3. **Problem-Solving Strategies**

How does your family approach finding and implementing solutions to problems involving your child?

Are there specific strategies or methods (e.g., planning, discussing) that have helped you overcome challenges?

IV. Open-Ended Questions

1. What do you consider to be the biggest challenge in raising and educating your child?
2. What kind of support do you wish to see from society or policy?
3. Do you have any advice or experiences you'd like to share with other families in similar situations?

Closing:

Thank you very much for sharing your experiences and insights. Your input is invaluable to our research. If we have any follow-up questions, we will contact you. Likewise, feel free to reach out to us if you have any additional thoughts. We wish you and your family all the best!

File S2.

Consolidated Criteria for Reporting Qualitative Studies (COREQ): 32-Item Checklist

No.	Item	Guide questions/description	Yes, page No. /No /NA
**Domain 1: Research Team and Reflexivity**			
**Personal Characteristics**			
1.	Interviewer/facilitator	Which author/s conducted the interview or focus group?	5-6
2.	Credentials	What were the researcher's credentials? e.g., PhD, MD	5-6
3.	Occupation	What was their occupation at the time of the study?	5-6
4.	Gender	Was the researcher male or female?	5-6
5.	Experience and training	What experience or training did the researcher have?	5-6
**Relationship with Participants**			
6.	Relationship established	Was a relationship established prior to study commencement?	4-6
7.	Participant knowledge of the interviewer	What did the participants know about the researcher? e.g., personal goals, reasons for doing the research.	4-6
8.	Interviewer characteristics	What characteristics were reported about the interviewer/facilitator? e.g., bias, assumptions, reasons and interest in the research topic.	4-6
**Domain 2: Study Design**			
**Theoretical Framework**			
9.	Methodological orientation and theory	What methodological orientation was stated to underpin the study? e.g., grounded theory, discourse analysis, ethnography, phenomenology, content analysis.	3-4
**Participant Selection**			
10.	Sampling	How were participants selected? e.g., purposive, convenience, consecutive, snowball.	5

11.	Method of approach	How were participants approached? e.g., face-to-face, telephone, mail, email.	6
12.	Sample size	How many participants were in the study?	5<br>Table 1
13.	Non-participation	How many people refused to participate or dropped out? Reasons?	NA
**Setting**			
14.	Setting of data collection	Where was the data collected? e.g., home, clinic, workplace	6
15.	Presence of non-participants	Was anyone else present besides the participants and researchers?	6
16.	Description of sample	What are the important characteristics of the sample? e.g., demographic data, date.	5<br>Table1
**Data Collection**			
17.	Interview guide	Were questions, prompts, guides provided by the authors? Was it pilot tested?	6<br>Supp.
18.	Repeat interviews	Were repeat interviews carried out? If yes, how many?	NA
19.	Audio/visual recording	Did the research use audio or visual recording to collect the data?	6
20.	Field notes	Were field notes made during and/or after the interview or focus group?	6
21.	Duration	What was the duration of the interviews or focus group?	6
22.	Data saturation	Was data saturation discussed?	6
23.	Transcripts returned	Were transcripts returned to participants for comment and/or correction?	6
**Domain 3: Analysis and Findings**			
**Data Analysis**			
24.	Number of data coders	How many data coders coded the data?	6
25.	Description of the coding tree	Did authors provide a description of the coding tree?	6
26.	Derivation of themes	Were themes identified in advance or derived from the data?	6

27.	Software	What software, if applicable, was used to manage the data?	6
28.	Participant checking	Did participants provide feedback on the findings?	6
**Reporting**			
29.	Quotations presented	Were participant quotations presented to illustrate the themes / findings? Was each quotation identified? e.g., participant number.	7-12
30.	Data and findings consistent	Was there consistency between the data presented and the findings?	7-12
31.	Clarity of major themes	Were major themes clearly presented in the findings?	7-12
32.	Clarity of minor themes	Is there a description of diverse cases or discussion of minor themes?	7-12
